# Mechanisms of Hepatitis C Virus Escape from Vaccine-Relevant Neutralizing Antibodies

**DOI:** 10.3390/vaccines9030291

**Published:** 2021-03-20

**Authors:** Rodrigo Velázquez-Moctezuma, Elias H. Augestad, Matteo Castelli, Christina Holmboe Olesen, Nicola Clementi, Massimo Clementi, Nicasio Mancini, Jannick Prentoe

**Affiliations:** 1 Copenhagen Hepatitis C Program (CO-HEP), Department of Immunology and Microbiology, Faculty of Health and Medical Sciences, University of Copenhagen, 2200 Copenhagen, Denmark; rodrigo.velazquez.moctezuma@regionh.dk (R.V.-M.); elias.augestad@regionh.dk (E.H.A.); fpk237@sund.ku.dk (C.H.O.); 2 Department of Infectious Diseases, Hvidovre Hospital, 2650 Hvidovre, Denmark; 3 Laboratory of Microbiology and Virology, Università “Vita-Salute” San Raffaele, 20132 Milano, Italy; castelli.matteo@hsr.it (M.C.); clementi.nicola@hsr.it (N.C.); clementi.massimo@hsr.it (M.C.); mancini.nicasio@hsr.it (N.M.)

**Keywords:** hepatitis C virus, antibody escape, B-cell vaccine, virus neutralization

## Abstract

Hepatitis C virus (HCV) is a major causative agent of acute and chronic hepatitis. It is estimated that 400,000 people die every year from chronic HCV infection, mostly from severe liver-related diseases such as cirrhosis and liver cancer. Although HCV was discovered more than 30 years ago, an efficient prophylactic vaccine is still missing. The HCV glycoprotein complex, E1/E2, is the principal target of neutralizing antibodies (NAbs) and, thus, is an attractive antigen for B-cell vaccine design. However, the high genetic variability of the virus necessitates the identification of conserved epitopes. Moreover, the high intrinsic mutational capacity of HCV allows the virus to continually escape broadly NAbs (bNAbs), which is likely to cause issues with vaccine-resistant variants. Several studies have assessed the barrier-to-resistance of vaccine-relevant bNAbs in vivo and in vitro. Interestingly, recent studies have suggested that escape substitutions can confer antibody resistance not only by direct modification of the epitope but indirectly through allosteric effects, which can be grouped based on the breadth of these effects on antibody susceptibility. In this review, we summarize the current understanding of HCV-specific NAbs, with a special focus on vaccine-relevant bNAbs and their targets. We highlight antibody escape studies pointing out the different methodologies and the escape mutations identified thus far. Finally, we analyze the antibody escape mechanisms of envelope protein escape substitutions and polymorphisms according to the most recent evidence in the HCV field. The accumulated knowledge in identifying bNAb epitopes as well as assessing barriers to resistance and elucidating relevant escape mechanisms may prove critical in the successful development of an HCV B-cell vaccine.

## 1. Introduction

Hepatitis C virus (HCV) infection is one of the leading causes of liver-related disease and liver transplantation in developed countries [1,2]. The World Health Organization (WHO) estimates that at least 70 million people are chronically infected with HCV worldwide and that approximately 400,000 people die every year from complications of long-term HCV infection, such as cirrhosis and hepatocellular carcinoma [3].

HCV is a single-strand positive-sense RNA virus (Baltimore classification, group IV) belonging to the *Hepacivirus* genus of the *Flaviviridae* family [4]. HCV has been classified into 8 genotypes with a variability of about 30% at the nucleotide and amino acid levels and more than 80 subtypes with a variability of about 20% at the nucleotide level [5,6,7,8]. The virus genome encodes a single polyprotein that is processed into 3 structural proteins, 6 nonstructural proteins and the viroporin, p7 [9]. The structural proteins are, by definition, components of the virus particle, which consists of the RNA genome encapsulated by the core protein, which is further enveloped in a lipid bilayer of cellular origin in which the transmembrane glycoprotein complex, E1/E2, is embedded. The nonstructural proteins serve critical functions in viral replication, translation, morphogenesis, and release into the extracellular environment [9].

In May 2016, WHO proposed the “Global Health Sector Strategy on Viral Hepatitis, 2016–2021”, with the intention of eliminating viral hepatitis as a public health problem by 2030 and naturally focusing on the main causative agents: hepatitis B and C. However, this goal has proven difficult to achieve for HCV without the development of a prophylactic vaccine. HCV treatment regimens have reached cure rates of around 95% in clinical trial and cohort studies [3,10]. However, treatment does not protect from reinfection, and high drug cost remains a barrier in most developing countries [11]. Furthermore, WHO estimates that, globally, only 19% of chronic HCV carriers have been diagnosed [3] due to lack of symptoms in early stages of the infection and inadequate pro-active screening efforts. 

### 1.1. E1/E2 as a Target of Neutralizing Antibodies in Vaccine Development 

Around 70% of HCV-infected patients will develop chronic infection [3]. The remaining 30% of people acutely infected with HCV spontaneously clear the infection without treatment [3,12], and around 80% of these patients are able to control and clear HCV reinfection [13]. Acute self-limited infection correlates with the presence of neutralizing antibodies (NAbs) in the early phase of HCV infection [14,15,16,17]. Moreover, the rapid induction of NAbs has been related with virus clearance [16] and pre-infusion of NAbs has been shown to prevent HCV infection in chimpanzees [18,19] and human liver chimeric mice [20,21,22,23].

**Figure 1:**
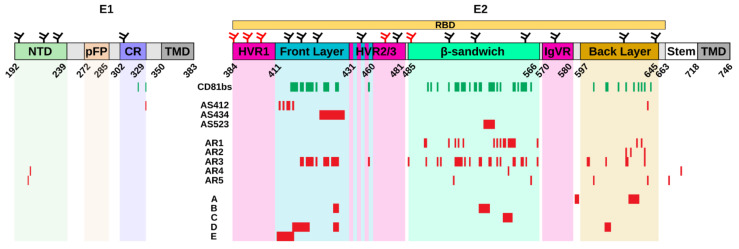
**Schematic representation of the primary E1/E2 sequence, including functional regions and epitope clusters.** Domain legends not defined in the main text: NTD (N-terminal domain), pFP (putative fusion peptide), CR (conserved region), CD81bs (CD81 binding site). CD81bs and conformational epitopes defined as in Gopal et al. 2017 [47]. Residues comprising each epitope are reported in red, residues involved in the interaction with CD81 in green. N- and O-glycosylation sites are depicted as black and red branched forks, respectively.

**Figure 2:**
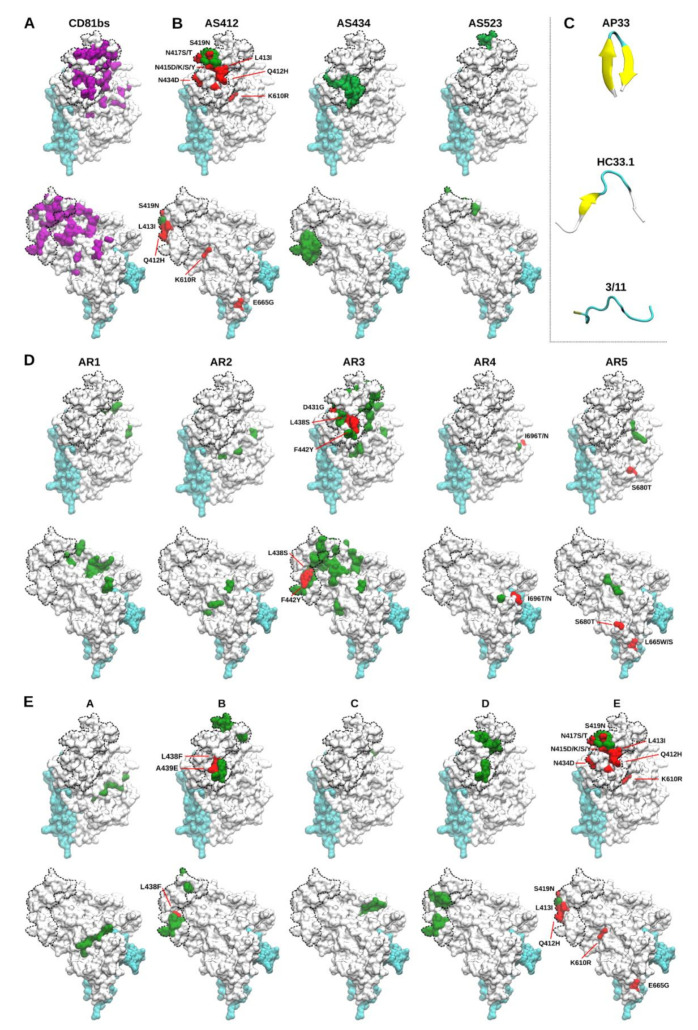
**Structural mapping of E1/E2 CD81bs, epitope clusters, and escape mutations.** (**A**) Residues participating or affecting the binding to CD81 according to Gopal et al. 2017 [47] are represented in purple. The CD81bs originally defined by competition assays, mutagenesis, and electron microscopy is indicated as a dashed line in all structures. (**B**) Depicts antigenic sites AS412, AS434, and AS523. (**C**) Solved crystal structures of AS412 peptide in complex with AP33 (PDB ID: 4GAG), HC33.1 (PDB ID: 4XVJ), and 3/11 (PDB ID: 4WHY). Yellow arrows depict β-sheet secondary structures. (**D**) Depicts antigenic regions 1–5. (**E**) Depicts antigenic domains A–E. Epitopes are consistently depicted in green, position of described escape mutations in AS412 (412, 413, 415, 417, 419, 434, 610, 665), AR3A (431, 438, 442), AR4A (696), AR5A (665 and 680), domain B (438, 439), and domain E (412, 413, 415, 417, 419, 434, 610, 665) are shown in red (see Table 1 for escape mutation overview). All epitope clusters are mapped on the E1/E2 ectodomain structural model described in Castelli et al. 2017 [76]. Escape mutations in E1 (A349D for AR5A and M345T for AR3A) are not shown as these were not included in the model.

The E1/E2 complex is the principal target of NAbs and, consequently, an attractive antigen for B-cell-based vaccine designs [24]. E1/E2 is important for receptor interactions during viral entry as well as membrane fusion with the host cell and viral particle release from infected cells [25]. During virus entry, the initial attachment occurs by interactions between the HCV-associated apolipoproteins, cell surface proteoglycans, and the scavenger receptor class B type I (SR-BI) [26,27]. Following attachment, E1/E2 interacts with the tetraspanin CD81, and the virions are translocated to the tight junctions where the HCV–CD81 complex interacts with claudin-1 and occludin, facilitating internalization via clathrin-mediated endocytosis [28,29,30]. E1/E2 is highly glycosylated, and several studies have shown roles of E1/E2 N-linked glycans in proper protein folding, virus entry, and protection from NAbs [31,32]. The 278 N-terminal amino acids of E2 constitute the receptor binding domain (RBD), which is followed by the stem region that connects the RBD with the C-terminal transmembrane domain (TMD) (Figure 1). The RBD contains amino acids critical for binding to the HCV co-receptor, CD81 (Figure 1 and Figure 2A), along with conserved epitopes that are the target of NAbs with broad neutralization activity (bNAbs) and, therefore, it has been suggested that most bNAbs inhibit HCV infection by blocking CD81 binding [24,33]. However, it is worth noting that the introduction of an affinity tag in E2 left the virus susceptible to neutralization by tag-specific antibodies, likely because the bulk of a bound antibody can interfere sterically with protein function [34]. The RBD contains 4 regions with high genetic diversity named hypervariable regions (HVR), including HVR1 (384–410), HVR2 (461–481), HVR3 (431–466), and the intergenotypic variable region (IgVR) (570–580) (Figure 1). In most isolates, HVR1 corresponds to the 26–27 N-terminal amino acids of E2. Several studies have shown that HVR1 modulates the interaction of E1/E2 with entry co-receptors such as CD81 and SR-BI in addition to preventing antibody neutralization by acting as an immune decoy, perhaps by hindering access to conserved NAb epitopes. This latter effect likely functions through an incompletely understood mechanism involving the stabilization of “closed” antibody-resistant E1/E2 states [35,36,37,38,39,40]. As is discussed in the following sections, several studies suggested that E1/E2 exists in an equilibrium between and open (antibody-sensitive) and closed (antibody-resistant) states [41,42,43]; however, there are currently no published structures to support this hypothesis and, as such, they cannot be adequately represented here. HVR1-deleted HCV recombinants typically have greatly increased susceptibility to NAbs [36,38,39], which has also been verified in vivo [37]. The remaining HVRs have received less attention, but it seems that HVR2 and IgVR are essential in E1/E2 oligomerization, HCV particle formation, and virus entry [44,45], and that HVR3 may play a role in binding to co-receptors during virus entry [46]. 

### 1.2. E1/E2 Immunogenic Epitope Clusters

The nomenclature of E1/E2 immunogenic clusters has been recently reviewed [48,49,50,51]. Zhang et al. described the antigenic sites (linear epitopes) 412–419 (AS412) and 434–446 (AS434) [52,53] (Figure 1 and Figure 2B). Some antibodies against AS412 and AS434 have shown broad neutralization activity, and these antigenic sites have been proposed as useful targets in HCV vaccine designs [48,54]. AS412 sits immediately downstream of HVR1 and is highly conserved. This conservation extends to residues that are important for CD81 binding (W420 and H421 [33,55]) as well as the N-linked glycosylation sites N417 and N423. NAbs against AS412 include 3/11 (anti-E2 murine monoclonal antibody (MAb) [56], AP33 (anti-E2 murine MAb) [57], HC33-related antibodies (anti-E2 human MAb (HMAb)) [58], H77.39 (anti-E2 murine MAb) [59], and HCV1 (anti-E2 HMAb) [60]. Notably, alanine substitution at positions 329, 613, and 624, outside the AS412 itself, reduced AP33 and HCV1 binding to E1/E2, suggesting that AS412 accessibility was allosterically modified [47]. Structural studies of E2 have described AS412 as a disordered region with an inherent high degree of structural flexibility [61,62,63]. Linear peptides corresponding to AS412 have been observed to bind to different NAbs in three distinct conformations: β-hairpin (in which the peptide folds back on itself), extended, and an intermediate V-shaped conformation [64,65,66,67,68,69,70] (Figure 2C). The structural analysis of AS412 bound to bNAbs AP33 and HCV1 showed a β-hairpin conformation displaying a hydrophilic face and a hydrophobic face on opposite sides of the hairpin, in which the NAbs predominantly interact with the amino acids L413 and W420 [66,67]. The V-shaped conformation of AS412 has been observed in structural analysis of a peptide containing AS412 in complex with bNAbs HC33.1, HC33.4, and HC33.8 [68,69]. The residues 414 and 415 adopt an antiparallel β-sheet with the variable region of the heavy chain of HC33.1, while the rest of AS412 assumes a coil conformation [68]. The extended conformation of AS412 was observed in complex with the poorly neutralizing antibody, 3/11 [70]. The low neutralizing capacity of 3/11 may indicate that this conformation of AS412 has the least relevance for mature virus particles. AS434 is a linear epitope composed of highly conserved residues important for E2 binding to CD81 [71,72]; however, AS434 is recognized by both bNAbs and non-neutralizing antibodies [52,72]. Non-neutralizing antibodies against AS434 have been shown to disrupt the interaction of bNAbs with AS412 [52]; on the other hand, antibodies against AS434 include the human HC84-related bNAbs with potent neutralization activity against HCV genotypes 1–6 [72,73]. Finally, a third linear epitope composed of residues 523–538 (AS523) has been shown to be involved in the neutralizing face of E2 together with the AS412 and AS434 [61] (Figure 1 and Figure 2B). Similarly to AS412 and AS434, AS523 contains highly conserved residues necessary for E2 interaction with CD81 [33]. To our knowledge, no antibodies exclusively targeting AS523 have been described. Antibodies that target epitopes which contain AS523 include 1:7, A8 (anti-E2 HMAbs) [74], H77.31, H77.36 (anti-E2 murine MAbs) [59], and CBH5 (anti-E2 HMAb) [75].

In addition to the antigenic sites described above, other groups have defined other systems of nomenclature to classify continuous and noncontinuous epitope clusters on E1/E2 mainly through antibody binding competition assays. Law et al. used a phage display library to isolate antibodies from a patient infected with HCV genotype 1 [22,23]. Using cross-competition assays, the epitopes recognized by these antibodies were classified into 5 antigenic regions (AR) [22,23] (Figure 1 and Figure 2D). AR1 antibodies have poor neutralizing activity and only bind to E2 from HCV genotype 1 [22]. AR2 is distal to the RBD of E2, and antibodies against AR2 were initially thought to be specific against HCV genotype 1. However, we found that low or non-existent neutralization by AR2-specific antibodies was due to a high level of epitope protection by HVR1 as we observed effective AR2A neutralization activity against HVR1-deleted viruses from genotypes 1 to 3 [22,38]. AR3 overlaps with the RBD and is highly conserved between HCV genotypes, showing broad neutralization activity against HCV genotypes 1–6 [22]. Antibodies against AR4 and AR5 only recognize folded E1/E2 complexes and have shown broad neutralization activity across HCV genotypes 1–6 [23]. Finally, AR3 and AR4 antibodies have been shown to be capable of protecting an HCV-permissive humanized mouse model from HCV challenge [77].

A different classification of epitope clusters was proposed by Keck et al. By using antibody cross-competition with human antibodies, they identified 4 conformational epitope clusters in E2, denominated antigenic domains A to D, and one linear cluster designated antigenic domain E [72,78,79], which is equivalent to AS412 [33,78] (Figure 1 and Figure 2E). These antigenic domains do not represent protein domains but, rather, clusters of E2 immunogenicity. Antibodies against domains B to E have shown broad neutralization against diverse HCV genotypes, whereas antibodies against domain A (residues 581–584 and 627–633) cannot neutralize HCV infection nor block CD81 binding [79,80,81,82]. Domain B (residues 441–443 and 529–535) and domain D (residues 420–428, 441–443 and 616) overlap with AR3, and both include important residues for CD81 interaction [22,58]. Domain B is highly immunogenic as antibodies against domain B are commonly found in infected patients [17,83]. NAbs against domain C (residues 544–549) overlapped with AR5 during immune competition analysis and epitope mapping. However, while AR5 NAbs only recognize folded E1/E2, domain C NAbs can also bind to E2 alone [23]. Thus, while HCV diversity is high, several highly conserved epitope clusters exist, indicating a high degree of functional constraint across HCV genotypes. The successful targeting of these clusters is likely to be instrumental in developing an HCV vaccine.

## 2. HCV Variability and Neutralization Escape

An important challenge in developing an HCV vaccine is the virus’ ability to evade antibody neutralization by mutational escape [24,84]. The lack of proofreading capacity of the HCV RNA-dependent RNA polymerase and the high viral replication rates result in a continually evolving within-host population of divergent viral quasispecies [85,86]. Although the generation of quasispecies is a random process, the selective pressure of the immune system results in the gradual appearance of escape mutants and genetic polymorphisms associated with resistance to T- and B-cell immune targeting [87,88]. In the following sections, we will summarize HCV studies of antibody neutralization in vitro, escape mutations, and genetic polymorphisms associated with antibody evasion.

## 3. Development of In Vitro Systems to Study Antibody Neutralization and Escape

For many years, the study of HCV was hampered by the lack of robust in vitro cell culture systems. The generation of HCV pseudoparticles (HCVpp) allowed the study of virus entry, including the role of putative entry co-receptors and the neutralization capacity of NAbs [89]. However, the HCVpp model system has several shortcomings, including the fact that it did not permit the study of other aspects of the viral cycle or of virus escape through antibody- or antiviral-induced culture adaptation.

The study of the full viral cycle was made possible with the development of full-length HCV cell culture systems (HCVcc), which permit the incorporation of diverse HCV structural protein sequences and initially depended on the highly replication-competent genotype 2a isolate, JFH1 [90,91,92,93]. These culture systems have been used to study the full viral cycle and the effect of NAbs and antivirals on viral infectivity and adaptation in vitro. In addition, they have formed the basis for various ways to examine escape and the barrier to resistance of HCV-specific bNAbs as reviewed in the following section.

## 4. Advancements in the Study of HCV Antibody Escape

### 4.1. In Vitro Escape Studies Using HCVcc

The development of HCVcc systems has enabled in vitro study of antibody escape and assessment of barriers to resistance for important bNAbs. This permits the prediction of future challenges with the emergence of potentially vaccine-resistant viruses and suggests ways in which to minimize such concerns [94]. However, many HCV isolates are either highly fit or naturally resistant to NAbs, both of which enable the virus to spread in culture in the presence of high concentrations of NAb without acquiring escape mutations [95,96]. Circumventing this problem has typically been done by only studying NAb escape using HCVcc that incorporate highly NAb-sensitive HCV isolates. Gal-Tanamy et al. exposed the highly bNAb-sensitive HCVcc, Hj3-5 (isolate H77, genotype 1a), to six sequential rounds of neutralization with the bNAb, AP33 (targeting AS412), followed by amplification in naive cultured cells [97]. Through this methodology, they identified the substitutions N415Y (E2) and E655G (E2). Reverse genetic studies showed that N415Y dramatically increased AP33 neutralization resistance, and this effect was greatly increased in the presence of E655G. Interestingly, E655G alone only conferred a modest increase in AP33 resistance. Keck et al. studied the barrier to resistance of antibodies against the antigenic domain B, such as CBH-2 and HC-11, and the antigenic domain E antibody, HC33.1, by using the highly bNAb-sensitive HCVcc JFH1 (genotype 2a) [78,80]. For CBH-2 and HC-11, they serially passaged JFH1 in the presence of increasing concentrations of antibody, starting at half the IC_50_ to 10–50 times the IC_50_ [80]. Following treatment with HC-11, they identified the substitution L438F (E2) that induced bNAb-specific resistance but also decreased viral fitness. For CBH-2, they identified the substitution A439E (E2), which conferred CBH-2-specific resistance without affecting fitness. For the antibody HC33.1, they employed a similar approach to identify multiple escape pathways [78]. HCVcc that escaped HC33.1 treatment contained the substitution N417T (E2) in combination with additional single or double substitutions in E2 that conferred different degrees of antibody resistance. N417T alone did not confer resistance against HC33.1 but increased HCVcc fitness and sensitivity to neutralization. N417T, in combination with N434D (E2) or K610R (E2), conferred low bNAb-specific resistance. HC33.1 resistance was higher with N417T combined with S419N (E2), while the highest effect was conferred by the combination N417T with L413I (E2) [78].

The escape studies mentioned above were limited to highly sensitive HCVcc, likely due to difficulties in working with naturally bNAb-resistant isolates that would require a considerable amount of antibody as evidenced by Pantua et al. and Gu et al., who serially cultured the bNAb-resistant HCVcc, Jc1 (genotype 2a) until it spread in the presence of increasing concentrations of AP33 (up to 200 μg/mL or ~40 times of the IC_90_ concentration) or another AS412-specific antibody Mab24 (up to ~200 μg/mL or ~20 times of the IC_90_ concentration) [64,98]. Through this approach, they identified the substitution N415D (E2) as well as N417S and N417T (E2). These substitutions increased resistance to AP33, Mab24, and other antibodies against AS412, such as HCV1. Further analysis showed that N417S and N417T shifted the N-linked glycosylation site from N417 to N415, which contributed to AP33 and Mab24 resistance. Structural analysis showed that HCV1 and AP33 bind to a similar AS412 β-hairpin structure, which is glycosylated at position N417 and N423 [66,67]. While glycans at N417 and N423 are pointing away from the epitope/paratope interface, the glycan introduced at N415 by N417S/T substitutions is buried in this interface and, thus, the glycan shift to position N415 sterically obstructs HCV1, AP33, and Mab24 binding. Remarkably, the N417 to N415 glycan shift increased sensitivity against HC33.1, which recognizes another conformation of AS412 [78]. Structural studies have shown that HC33.1 interacts with a V-shape conformation of the linear epitope, where the glycan at N415 is placed outside the interface and flanking HC33.1 [68]. Thus, the presence of a glycan at N415 may assist in the interaction with E2, perhaps explaining the increased affinity of HC33.1 with AS412.

Finally, a different approach was used by Duan et al., in which they introduced naturally occurring substitutions or polymorphisms observed at positions 412–426 of genotypes 1 and 2 into the HCVcc, J6/JFH1 [99]. Thus, they identified the resistance substitution Q412H that conferred resistance against AS412 bNAbs from chimpanzees vaccinated with HCV recombinant E1/E2 but not against an immunoglobulin preparation composed of polyclonal antibodies from HCV patients.

### 4.2. Validation of AS412 Escape In Vivo

In addition to in vitro escape studies as outlined above, escape substitutions have been found during in vivo antibody treatment. Escape variants against the antibody HCV1, targeting AS412, have been identified in both chimpanzee and human studies. Chimpanzees were infected with HCV and treated with HCV1 during the acute HCV infection phase, or after they developed chronic infection [18]. Following 42 days of HCV1 treatment, the escape substitutions N415K or N415D (E2) emerged. Similarly, the escape substitution N417S was identified in emerging viruses after a single dose of HCV1 antibody to a chronic HCV-infected chimpanzee. In humans, the capacity of HCV1 to clear HCV infection after liver transplantation was tested in a clinical trial by treating infected patients with multiple doses of HCV1 [100,101]. While not all patients were able to clear HCV infection, a delay in viral rebound was observed among all treated subjects. Like the chimpanzee studies, emerging viruses exhibiting substitutions N415K/D/S and N417S were identified in antibody-resistant variants found in patient sera. As mentioned above, the described substitutions at position N415 and N417 reduce E2 binding and neutralization sensitivity to HCV1 [18,64,97].

In vivo escape studies have been focused on bNAbs that recognize AS412 and, thus, more information is needed for bNAbs targeting other vaccine-relevant epitopes. Previously, Farci et al. identified HVR1 escape mutants by incubating an H77 virus inoculum with serum containing NAbs against HVR1 and infecting chimpanzees with the virus/antibody mix [102]. Although they showed that chimpanzees can be used as an in vivo model to study antibody escape, biomedical research in chimpanzees is nowadays banned in most developed countries. Small animal infection models would be an attractive alternative using, for example, HCV-permissive human liver chimeric mice to identify escape substitutions following antibody treatment in vivo. An example of this approach is the identification of the substitution D476G (E2), which was observed during treatment of HCV-infected mice with polyclonal antibodies from a chronic-phase HCV patient serum termed H06 [37]. In vitro testing showed that the emerging substitution, D476G, conferred broad, low-level antibody resistance against both H06 antibodies as well as the three bNAbs AR3A, AR4A, and AR5A, showing the feasibility of using this mouse model to study bNAb escape in vivo. Importantly, the observed overlaps in emerging escape substitutions between in vitro and in vivo studies also supports the use of cell culture approaches to study HCV antibody escape.

### 4.3. Induction of Antibody Escape in the NAb-Sensitive HVR1-Deleted HCVcc

While numerous important in vitro studies have assessed escape for HCV bNAbs, the studies have, apart from Pantua et al. and Gu et al., been based on treating broadly bNAb-sensitive HCVcc, introducing a potential bias in the results. Importantly, the studies have also focused on single isolates of HCV, ignoring the potential differences in escape, which are likely given the high sequence diversity of the virus. However, the study of inherently NAb-resistant HCV isolates is made difficult by the high expenditure of antibody needed to induce escape, and this problem is naturally compounded by trying to study escape for multiple isolates. Accordingly, we tried to induce resistance by passaging the moderately bNAb-resistant HCVcc, H77/JFH1 (genotype 1a), and the resistant HCVcc, J6/JFH1 (genotype 2a), in the presence of high concentration of the bNAb, AR5A [95], but found that J6/JFH1 escaped treatment without acquiring envelope protein substitutions. In contrast, H77/JFH1 acquired the substitution A349D (E1), which conferred broad, low-level bNAb resistance. In separate studies, we tried to generate escape by treating H77/JFH1 with higher amounts of the bNAbs AR3A and AR4A [42,103]. For AR4A, we identified the substitution M345T (E1), but this substitution only increased fitness and did not affect AR4A resistance [103]. Surprisingly, treatment of H77/JFH1 with AR3A antibody also led to the appearance M345T, which induced low-level AR3A-specific resistance in addition to its effects on fitness, implying that M345T could be allosterically modifying access to the AR3A E2 epitope [42]. These studies confirmed how difficult it is to induce escape even in moderately NAb-resistant HCVcc, which can spread in culture without developing NAb escape substitutions or may merely gain substitutions that increase fitness.

**Table 1:** Advancements in the study of HCV antibody escape.

Escape Substitutions	Antibody Studied	HCV Isolate	Location	Resistance Mechanism	Reference
**N415Y**	AP33	H77 genotype 1	E2; AS412, Domain E	Direct modification of the epitope	[97]
**97E665G**	AP33	H77 genotype 1	E2; AS412, Domain E	Indirect modification of the epitope	[97]
**L438F**	HC-11	JFH1 genotype 2	E2; Domain B	Direct modification of the epitope	[80]
**A439E**	CBH-2	JFH1 genotype 2	E2; Domain B	Direct modification of the epitope	[80]
**N417T/N434D**	HC33.1	JFH1 genotype 2	E2; Domain B	Indirect modification of the epitope	[78]
**N417T/K610R**	HC33.1	JFH1 genotype 2	E2; Domain B	Indirect modification of the epitope	[78]
**N417T/S419N**	HC33.1	JFH1 genotype 2	E2; AS412, Domain E	Direct modification of the epitope	[78]
**N417T/L413I**	HC33.1	JFH1 genotype 2	E2; AS412, Domain E	Direct modification of the epitope	[78]
**N415D**	AP33/Mab24	Jc1 genotype 2	E2; AS412, Domain E	Direct modification of the epitope (broad increase in antibody sensitivity)	[64,98]
**N417S/T**	AP33 (Mab24 only for N417S)	Jc1 genotype 2	E2; AS412, Domain E	Direct modification of the epitope (Glycan shift and broad increase in antibody sensitivity)	[64,98]
					
**Q412H**	Chimpanzees and patients serum enriched against AS412	J6 genotype 2	E2; AS412, Domain E	Direct modification of the epitope	[99]
**N415K/D**	HCV1 in vivo	H77 genotype 1	E2; AS412, Domain E	Direct modification of the epitope	[18]
**N417S**	HCV1 in vivo	H77 genotype 1	E2; AS412, Domain E	Direct modification of the epitope (Glycan shift)	[18]
**N415K/D/S**	HCV1 in vivo	genotype 1	E2; AS412, Domain E	Direct modification of the epitope	[100,101]
**N417S**	HCV1 in vivo	genotype 1	E2; AS412, Domain E	Direct modification of the epitope (Glycan shift)	[100,101]
**D476G**	HO6	J6 genotype 2	E2	Broad impact on antibody sensitivity	[37]
**A349D**	AR5A	H77 genotype 1	E1	Broad impact on antibody sensitivity	[95]
**M345T**	AR3A	H77 genotype 1	E2: AR3	Indirect modification of the epitope and increase of virus fitness	[42]
**L665W**	AR4A	H77 genotype 1	E2; AR5	Direct modification of the epitope	[95]
**L665S**	AR5A	J6 genotype 2	E2; AR5	Direct modification of the epitope	[95]
**S680T**	AR5A	J6 genotype 2	E2; AR5	Indirect modification of the epitope	[95]
**I696T/N**	AR4A	J6 genotype 2	E2; AR4	Direct modification of the epitope (I696N increased broad sensitivity to other antibodies)	[103]
**L438S**	AR3A	H77 genotype 1	E2; AR3	Direct modification of the epitope	[42]
**F442Y**	AR3A	H77 genotype 1	E2; AR3	Direct modification of the epitope	[42]
**D431G**	AR3A	J6 genotype 2	E2; AR3	Direct modification of the epitope (broad increase in antibody sensitivity)	[42]

In order to overcome this limitation, the studies of AR3A, AR4A, and AR5A NAb escape mentioned above also employed bNAb-sensitive HVR1-deleted variants of the studied HCVcc, namely H77/JFH1_ΔHVR1_ (genotype 1a) and J6/JFH1_ΔHVR1_ (genotype 2a) [42,95,103]. By passaging H77/JFH1_ΔHVR1_ in the presence of AR5A we identified the substitution L665W (E2), which caused high levels of AR5A-specific resistance [95]. Additionally, L665W increased AR5A resistance in a panel of diverse HCVcc, spanning genotypes 1 to 6, while decreasing virus fitness of genotypes 2, 3, 4, and 6. Interestingly, in a similar approach for J6/JFH1_ΔHVR1_, we identified the substitution L665S (E2), which only caused low-level AR5A resistance. However, in combination with another co-selected substitution, S680T (E2), this resistance was significantly boosted, suggesting a more complex resistance pathway and highlighting the importance of studying escape in multiple HCV isolates. L665S decreased AR5A sensitivity but reduced viral fitness, while the combination with S680T further increased AR5A resistance and compensated the fitness loss. Surprisingly, S680T alone increased AR5A sensitivity and did not affect fitness, suggesting a complex relationship between these two positions and the AR5A epitope. Similarly, we identified the substitution I696T (E2) in J6/JFH1_ΔHVR1_ that conferred AR4A-specific resistance, but we were unable to induce escape for H77/JFH1_ΔHVR1_ [103]. Additionally, we identified the resistance mutation I696N (E2), which reduced AR4A sensitivity of J6/JFH1_ΔHVR1_ but did not affect AR4A sensitivity of parental J6/JFH1. This was likely because I696N greatly increased broad bNAb sensitivity of the parental HCVcc. Finally, we identified the resistance mutations L438S (E2) and F442Y (E2) for H77/JFH1_ΔHVR1_, but they dramatically reduced fitness of the parental HCVcc that retained HVR1 [42]. Substitutions at position L438 and F442 have been shown to reduce binding to CD81 and, thus, it is possible that the loss of fitness is due to deficient CD81 engagement during entry [47,71]. For J6/JFH1_ΔHVR1_, we observed the substitution D431G (E2), which increased AR3A resistance, whereas it only marginally affected AR3A sensitivity of the parental J6/JFH1. Interestingly, D431G increased broad bNAb sensitivity in analogy to the AR4A escape substitution I696N.

## 5. Evidence for Different Mechanisms of HCV NAb Resistance

**Figure 3:**
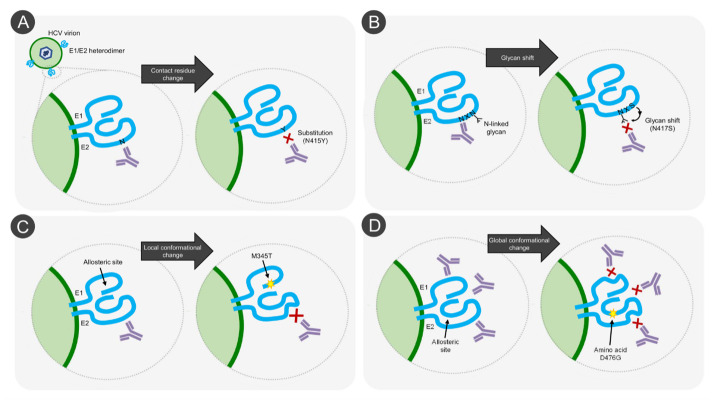
**Mechanisms of direct HCV escape from neutralizing antibodies.** (**A**) Contact residue change. Substitutions that directly alter a contact residue, whereby antibody–antigen affinity is reduced. (**B**) Glycan shift. Altered glycosylation can lead to HCV resistance to NAbs. The glycan masks the broadly neutralizing epitope on the viral glycoproteins, thus preventing neutralization of the viral particle. (**C**) Local allosteric change. Substitutions at positions not directly within the epitope can alter HCV sensitivity to a specific NAb by inducing local allosteric changes that alter specific NAb epitope accessibility. (**D**) Global allosteric change. Substitutions at positions not directly within the epitope can alter HCV sensitivity to a wide range of NAbs by inducing global allosteric changes that alter NAb epitope accessibility.

**Figure 4:**
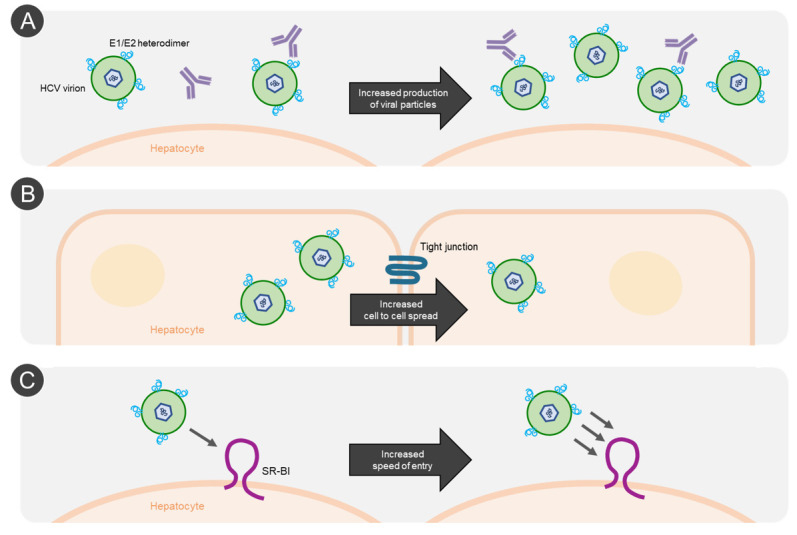
**Indirect HCV neutralizing antibody escape by increased viral fitness, cell-to-cell spread, and speed of entry.** HCV mutations can lead to (**A**) increased production of viral particles, thus increasing the number of infectious particles at any given concentration of antibody; (**B**) increased cell-to-cell spread, resulting in an increase in viral particles avoiding NAbs present in the extracellular environment; and (**C**) increased speed of entry, affording NAbs in shorter time to interact with their epitopes on the viral particles in the extracellular environment.

The escape studies reviewed above not only revealed important information about HCV bNAb epitopes and their barrier to resistance, but in many instances, they also shed light on distinct escape mechanisms employed by the virus. Specific changes in the epitopes (such as the substitution of an epitope/paratope contact residue) was induced by substitutions L413I (HC33.1), N415K/D/S/Y (AP33, HCV1), S419N (HC33.1), D431G (AR3A), L438F (HC-11), L438S (AR3A), A439E (CBH-2), F442Y (AR3A), I696T/N (AR4A), and L665W/S (AR5A), thus directly impacting the antibody–antigen interaction [42,64,95,97,103] (Figure 3A). A variation on the theme of substitutions that directly alter the epitope is exemplified by the glycan-shift resistance substitutions N417S or N417T, which shifts the N-linked glycosylation of N417 to position N415, thus interfering with binding of bNAbs AP33 and HCV1 [64] (Figure 3B). In addition to these more straightforward mechanisms of antibody escape, evidence also exists for epitope-specific allosteric effects causing bNAb resistance at distant sites, as substitutions that do not appear to be part of the epitope itself are able to specifically alter HCV sensitivity against individual bNAbs (Figure 3C). This is exemplified by E655G, that enhanced resistance against AP33 in the presence of N415Y, the E1 substitution M345T that increased AR3A resistance, or S680T that enhanced AR5A resistance in the presence of L665S but made the virus more susceptible to AR5A in the absence of L665S [95,97]. Such allosteric effects with a broader impact on NAb sensitivity (i.e., involving multiple epitopes) have also been described. Examples include escape substitutions such as, A349D, N415D, N417S, D431G, D476G, and I696N that affect resistance against a broad range of bNAbs [37,95,98,103] (Figure 3D). Notably, such resistance substitutions frequently also affect the SR-BI entry dependency of HCV. While the mechanism of these allosteric effects are still largely unknown, recent findings suggest that the E1/E2 envelope protein complex exists in a dynamic equilibrium between “open” (NAb-sensitive) and “closed” (NAb-resistant) states, as will be addressed in a subsequent section. Finally, mutations that increase HCV fitness also appear to increase the virus ability to spread in culture in the presence of bNAbs, likely by increasing the total number of HCV particles that remain infectious in the presence of a given concentration of antibody (Figure 4A) as well as possibly by augmenting cell-to-cell spread (Figure 4B) and/or increasing speed of entry, likely through an altered interaction with SR-BI (Figure 4C) [42,95,96,104,105]. The substitutions N417D for H77/JFH1_ΔHVR1_, I345V for J6/JFH1_ΔHVR1_, and N417T for JFH1 increased viral fitness without affecting bNAb sensitivity against the bNAbs used to induce escape [78,95]. It is important to emphasize that a single substitution can have more than one of these effects or have different effects in different virus variants. For example, M345T (E1) induced epitope-specific allosteric resistance against AR3A while also increasing fitness [42].

## 6. Both Naturally Occurring and Cell Culture Adaptive Envelope Substitutions Regulate Broad HCV Neutralization Sensitivity

Similarly to the E2 substitutions, D431G and I696N, culture adaptation studies of HCV have identified a number of envelope protein substitutions that increase broad NAb sensitivity [104,105,106,107,108]. Similarly, naturally occurring envelope protein substitutions that affect broad NAb sensitivity have been identified in diverse HCV genotypes and subtypes. Natural variation in bNAb resistance across HCV isolates has been attributed to resistance-associated envelope polymorphisms (RAPs). Such polymorphisms have been identified through neutralization clustering and sequence analysis of panels of HCVpp displaying E1/E2 complexes of patient-derived sequences [109,110] and by reference alignment and phylogenetic analysis of genotype 1a sequences to find codons that vary deep in the phylogenetic tree [111]. Additionally, we recently identified RAPs by an in-depth reverse genetics approach, studying the sequence determinants of bNAb resistance between two HCVcc isolates of the same genotype, H77 and TN (both genotype 1a) [43]. This work was based on our previous finding that HCV isolates with highly diverse neutralization profiles become similarly sensitive to bNAbs targeting diverse conserved E1/E2 epitopes by the removal of HVR1, indicating that these epitopes were significantly more conserved than initially supposed [38]. Interestingly, we found that isolate differences in bNAb sensitivity are linked to extra-epitopic variation, specifically RAPs in the N-terminal part of E2 [43]. Several of these RAPs were found within positions 400–404 in the C-terminus of HVR1, that has been shown to accumulate mutations during chronic infection [112,113]. Although the effect of a single RAP is often minor, combinations of such polymorphisms can alter antibody sensitivity by as much as 2000-fold [43]. However, the effect of individual RAPs varies across bNAb epitopes [43], which opens up the possibility that HCV accumulates these polymorphisms to fine-tune accessibility of epitopes as they become targeted by the host immune system.

## 7. Broad Neutralization Sensitivity of HCV Is Regulated by Global Envelope Conformation Dynamics

Similarly to substitutions that increase broad NAb sensitivity as mentioned before (A349D, N415D, N417S, D431G, D476G and I696N), our recent finding that removal of N-linked glycans on E2 broadly modulated bNAb sensitivity (both positively and negatively) [32] indicates a mechanism involving indirect protection from bNAbs rather than direct steric shielding of specific neutralization epitopes on E1/E2, as was previously suggested [31]. Interestingly, the broad NAb sensitizing or protective effect of individual E2 glycans as well as the broad effect of some escape substitutions is absent when HVR1 is removed [32,42,103]. Thus, N-linked glycans, RAPs, and HVR1 seem to modulate cross-epitope bNAb sensitivity through a shared mechanism. We speculated whether this mechanism involved perturbation of global conformation dynamics of the HCV envelope glycoproteins. Such dynamics have been described using X-ray scattering of proteins in solution [114] and have been examined in flaviviruses such as dengue virus, West Nile virus, and Zika virus. Here, antibody binding, neutralization, and cryo-electron microscopy studies are providing convincing evidence of the existence of structurally dynamic virions where the envelope proteins exist in a dynamic equilibrium between “open” (neutralization-sensitive) and “closed” (neutralization-resistant) states (e.g., envelope protein breathing) [115,116,117]. Expanding on previous HCV studies showing temperature dependence of virus neutralization [41], we performed temperature-dependent neutralization experiments with various modified HCVcc using multiple NAbs, which indicated that a mechanism involving perturbation of global conformation dynamics of the HCV envelope glycoproteins is responsible for modulating bNAb sensitivity [32,43]. Thus, it seems likely that RAPs as well as N-linked glycans mediate their effects on NAb sensitivity by regulating an existing HCV global envelope protein equilibrium from “open” to “closed” states, in which the stability of the “closed” states depends on the presence of HVR1 [32,43].

### 7.1. Local Conformations of AS412 Correlate with Global States of E1/E2

E1 polymorphisms have so far not been linked to significant effects on antibody sensitivity [110]. Accordingly, polymorphisms that broadly affect neutralization sensitivity of HCV are located in different parts of E2 (mainly the N-terminal region), including position 400–405 in HVR1, AS412, immediately downstream of HVR1, the front-layer region (aa 421–459) and the central β-sheet (aa 526–569) [43,110,111]. These polymorphisms may cause local structural rearrangement which, in turn, seem capable of influencing global conformation dynamics of E1/E2 to alter broad bNAb sensitivity. However, the impact of RAPs on conformations of local E2 sites might be concealed by the inherent differences in the global availability of these sites on E1/E2. Thus, to begin addressing this possibility, we recently performed a comparison of relative neutralization sensitivity of antigen-binding fragments (Fabs) with the corresponding MAbs targeting AS412 based on the idea that Fabs, due to their smaller size, better penetrate “closed” more-resistant states of E1/E2 [43]. We compared MAb:Fab neutralization sensitivity of HCV to the antibodies HC33.4 and AP33, which recognize an extended V-shaped form and a β-hairpin-like conformation, respectively, of the conserved, but structurally flexible AS412 (Figure 2C). Interestingly, our data were consistent with the hypothesis that broadly protective polymorphisms increased the Fab vs. MAb potency of AP33, seemingly skewing the AS412 conformational space towards β-hairpin-like conformations [43]. As several regions of E2 are characterized by analogous conformational plasticity [118], similar phenomena would be of interest to test for other bNAbs.

### 7.2. bNAb Resistance Influences Entry Dependency on HCV Co-Receptors

HCV interactions with the tetraspanin CD81 were recently proposed to occur via two routes, either directly or with prior engagement of the co-receptor SR-BI [119], which offers a partial explanation for why HCV isolates do not seem to depend completely on SR-BI interaction for viral entry. Interestingly, we found that RAPs increased HCV entry dependency on SR-BI, and that this correlated closely with the propensity of the virus to interact with CD81, suggesting a role of SR-BI during virus entry in mediating transitions from “closed”, bNAb-resistant conformational states of E1/E2 to “open” bNAb-sensitive states, the latter of which are primed for CD81 engagement [43]. This provides an attractive mechanistic explanation for the proposed existence of SR-BI dependent and independent routes of infection. Consequently, the positive effect of increased RAP-mediated bNAb protection might come at the cost of a more complicated entry pathway, which could help explain why HCV patient isolates present such a variety of RAP compositions.

## 8. Conclusions and Future Directions

Modulation of bNAb sensitivity by envelope protein escape substitutions, RAPs, HVR1, and N-linked glycans is not fully recapitulated on soluble E2 nor on cell-associated/extracted E1/E2 protein [32,42,43,110,120], suggesting a dearth in relevant models for studying antibody–E1/E2 interactions at a molecular level. This is possibly due to the importance of interactions within higher-order structural forms of the E1/E2 heterodimer on the virion surface [121,122], or because of a lack of virus-associated lipoproteins, as exemplified by the fact that high-density lipoprotein (HDL) and ApoE have been shown to increase differences in neutralization sensitivity [123,124,125].

The extensive efforts to understand HCV E1/E2 immunogenicity and, particularly, immunogenic epitope clusters, has permitted the identification of important HCV epitopes and antibodies useful for rational vaccine design. Such designs should ideally induce potent bNAbs against epitopes with high barriers to resistance in order to prevent the generation of vaccine-resistant variants. This phenomenon of vaccine resistance is observed for HBV, and given the fact that HCV is an intrinsically much more variable virus, such problems would be expected to be compounded [94]. A more salient comparison may be the emergence of vaccine-resistant variants for the RNA virus, SARS-CoV-2, which is the global health crisis emergent in 2020. Similarly to HCV E1/E2, the spike protein of SARS-CoV-2 has been found to accumulate escape mutations under the selective pressure of NAbs [126]. Further, many of the escape mutations identified in vitro have already been observed in circulating human isolates [126,127]. Although it is not clear how antibody escape would affect vaccine-induced immunity against SARS-CoV-2, the knowledge generated by studying escape would be an important tool for future vaccine designs.

AR3A and AR4A seem to represent promising vaccine targets as the bNAbs directing against them are broadly neutralizing with high potency, protect in animal challenge models and show high barriers to resistance. In contrast, for studied bNAbs targeting AS412, domain B and E, and AR5, the barrier to resistance appears lower. However, escape has not been studied for the majority of HCV NAbs and it is possible that other broadly reactive NAbs with high barriers to resistance target these or other antigenic domains or regions. In addition, it should be noted that finding ways in which to target multiple conformations of E2, such as recently described E2 A and B conformations or even of the linear E2 epitope AS412, may hinder HCV escape for this conserved epitope [43,128].

The study of antibody escapes in vitro using culture infectious viruses and in vivo in permissive animal models remains the only reliable way to determine barriers to resistance for HCV NAbs. These studies will be critical in selecting vaccine antigens for the development of HCV vaccine candidates capable of inducing enduring cross-genotype protection from HCV chronicity and impede the emergence of HCV escape variants.

## Data Availability

Not applicable.
